# Identification of deletion-duplication in *HEXA* gene in five children with Tay-Sachs disease from India

**DOI:** 10.1186/s12881-018-0632-7

**Published:** 2018-07-04

**Authors:** Jayesh Sheth, Mehul Mistri, Lakshmi Mahadevan, Sanjeev Mehta, Dhaval Solanki, Mahesh Kamate, Frenny Sheth

**Affiliations:** 1 Biochemical and Molecular Genetics, FRIGE’s Institute of Human Genetics, FRIGE House, Satellite, Ahmedabad, Gujarat 380 015 India; 2 Medgenome Labs Pvt Ltd, Bangalore, India; 3 Usha Deep Hospital, Ahmedabad, Gujarat India; 4 Mantra Child Neurology & Epilepsy Clinic, Bhavnagar, Gujarat India; 5 Department of Pediatric Neurology, KLES Prabhakar Kore Hospital, Belgaum, Karnataka India

**Keywords:** Tay-Sachs disease, ß-hexosaminidase-A, *HEXA* gene; MLPA

## Abstract

**Background:**

Tay-Sachs disease (TSD) is a sphingolipid storage disorder caused by mutations in the *HEXA* gene. To date, nearly 170 mutations of *HEXA* have been described, including only one 7.6 kb large deletion.

**Methods:**

Multiplex Ligation-dependent Probe Amplification (MLPA) study was carried out in 5 unrelated patients for copy number changes where heterozygous and/or homozygous disease causing mutation/s could not be identified in the coding region by sequencing of *HEXA* gene.

**Results:**

The study has identified the presence of a homozygous deletion of exon-2 and exon-3 in two patients, two patient showed compound heterozygosity with exon 1 deletion combined with missense mutation p.E462V and one patient was identified with duplication of exon-1 with novel variants c.1527-2A > T as a second allele.

**Conclusion:**

This is the first report of deletion/duplication in *HEXA* gene providing a new insight into the molecular basis of TSD and use of MLPA assay for detecting large copy number changes in the *HEXA* gene.

## Background

Tay-Sachs disease (TSD) [MIM* 606869] is one of the common sphingolipid storage disorder in India [1]. It is a rare neurodegenerative lysosomal storage disorder (LSD) caused by a deficiency of ß-hexosaminidase-A (Hex-A) (HEXA; EC: 3.2.1.52) enzyme. It occurs due to the inability of Hex-A enzyme to cleave the terminal N-acetyl hexosamine residues from GM2 ganglioside due to a mutation in *HEXA* gene. As a result, GM2 ganglioside is accumulated in various tissues especially in neuronal cells instead of further metabolizing into GM3 gangliosides [2, 3]. The clinical phenotype varies widely with an acute infantile form of early onset leading to rapid neuroregression and early death to a progressive later onset form compatible with a longer survival [2].

The human *HEXA* gene is mapped on chromosomes 15q23-q24 with 35.56 kb spans, containing 14 exons [4]. As per HGMD (Human Gene Mutation Database), nearly 170 mutations have been reported so far in the gene that causes TSD; that include 130 single base substitutions, 29 small deletions, 6 small insertions, 2 indels and 1 large deletion of 7.6 kb (http://www.hgmd.cf.ac.uk/). Of these only 7.6 kb deletion is reported as a largest one in *HEXA* gene which covers 70% of infantile TSD cases in French Canadians [5].

Our earlier studies on Indian patients affected with TSD revealed various novel and known missense, nonsense, splice site mutation and frameshift mutations [6, 7]. In the present study, Multiplex Ligation-dependent Probe Amplification (MLPA) - based approach (MRC-Holland, P199-B) was used to investigate for the potential occurrence of large *HEXA* deletions/duplications in addition to common mutation(s) screening and bidirectional sequencing of *HEXA* gene.

## Methods

The present study was carried out as a part of National Taskforce multicentric project of Indian Council of Medical Research (ICMR) and Department of Health Research (DHR), Government of India. The present study has been approved by the institutional ethics committee in accordance with the Helsinki declaration. A written informed consent was obtained from the parents before enrollment.

### Patients

MLPA study was carried out in 5 enzymatically confirmed TSD patients for deletion/duplication analysis where disease causing mutation was not identified in the coding region of the gene and/or single disease causing allele was identified by common mutations screening and bi-directional sequencing of *HEXA* gene.

### Multiplex ligation-dependent probe amplification (MLPA) analysis of *HEXA* gene

The genomic DNA was isolated from whole blood using salting out method [8]. MLPA analysis was carried out using P199-B2 HEXA P probe mix (MRC-Holland, Amsterdam, The Netherlands) in cases where Sanger sequencing failed to identify any pathological variant. The procedure was carried out according to the manufacturer’s recommendations using100 ng of genomic DNA. It was denatured at 98 °C for 5 min and hybridized overnight at 60 °C with the SALSA probe mix P199-B2 (*HEXA* gene, exons 1-14). Samples were then treated for ligation for 15 min at 54 °C. The reaction was stopped by incubation at 98 °C for 5 min. Finally, PCR amplification was carried out with the specific SALSA FAM PCR primers. Amplification products were run on an ABI PRISM 3100 Genetic Analyzer (Applied Biosystems, USA). Copy number differences of various exons between test and control DNA samples were detected by analyzing the MLPA peak patterns.

## Results

Molecular analysis was carried out in 75 TSD cases with deficiency of Hex-A and normal Total-Hex enzyme activity. Of these, 70 TSD patients have been identified with both coding mutations in *HEXA* gene while in 3 patients only one coding mutation was detected and in 2 patients no coding mutation was identified. Hence, MLPA study was carried out in these 5 unrelated patients to rule out copy number changes where heterozygous and/or homozygous disease causing variant could not be identified in the coding region by sequencing *HEXA* gene.

Consanguinity was present in 1/5 (20%) families. The mean age at presentation was 13.8 months (±2.48). All the cases were classified as infantile as they were presented with seizures, cherry red spot on the fundus, exaggerated startle, hypotonia, brisk deep tendon reflexes and regression of learned skill. The CT/MRI study of the brain was available in 3/5 cases and showed characteristic findings of a decrease in thalami and decreased attenuation of basal ganglia isodense with white matter, and one case had dysmyelination. A significant deficiency of Hex A activity was observed in the leukocytes of all five patients. The geographic/ethnic background, age at onset, age at last observation, enzyme activities and the genotypes identified are shown in Table 1.

**Table 1:** Clinical, biochemical and molecular details of the Indian patients with Tay–Sachs disease

Patient ID	Age at diagnosis(Months/ Sex	NativeState	Cosan-guinity	Hex-A activity(MUGS) (nmol/hr./mg) = (x)	Total Hexactivity(MUG) = (y)	^a^ HexA % =(x/y) X 100	Genotypes		Phenotypes
							Nucleotide level (Allele fromFather/ Allele from Mother)	Protein level (Allele from Father/Allele from Mother)	
1	18/M	Gujarat	No	0.9	1292.1	0.07	Exon-2-3del/ Exon-2-3del	Not	Regression of milestone, cherry red spot,abnormal muscle tone, hyperacusis, seizures,abnormal MRI,
2	14/M	Gujarat	No	1.05	Not done	–	Exon-2-3del/ Exon-2-3del	Not applicable	Regression of milestone, cherry red spot,poor vision, abnormal muscle tone,hyperacusis, seizures, abnormal MRI, abnormalEEG
3	12/F	Gujarat	No	3.8	2185.7	0.17	c.1385A > T/Exon-1 deletion	p.E462V/ Not applicable	Regression of milestone, cherry red spot,abnormal muscle tone, seizures, hyperacusis,hearing impairment
4	13/F	Gujarat	No	2.5	1635	0.15	c.1385A > T/Exon-1 deletion	p.E462V/ Not applicable	Regression of milestone, hypotonia,hyperacusis, cherry red spot, abnormal MRI
5	13/ M	Karnataka	Yes	1.78	2198.2	0.08	c.1527-2 A > T/Exon-1 duplication	Not applicable	Regression of milestone, cherry red spot,hypotonia

Normal total-Hexosaminidase values using MUG substrate in our controls − 723 to 2700 nmol/hr./mg protein and normal Hex-A activity using MUGS substrate- 80 to 410 nmol/hr./mg

^a^The MUG/MUGS ratio for Hex A is 3.7:1 [10]

**Fig. 1:**
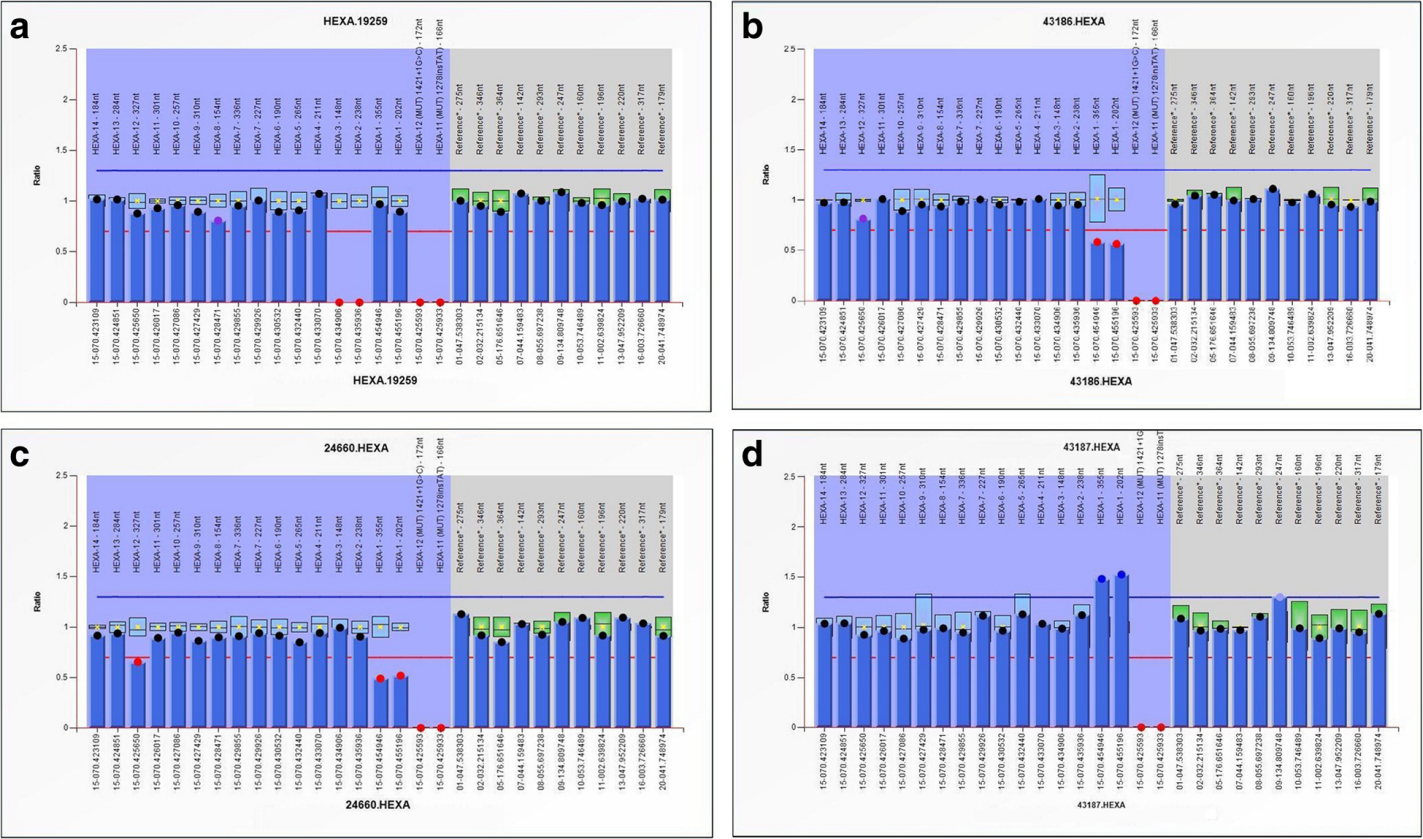
MLPA analysis of *HEXA* gene (**a**): homozygous deletion of exon 2 & 3; (**b**) & (**c**): heterozygous deletion of exon 1; (**d**): heterozygous duplication of exon 1

The MLPA analysis of *HEXA* gene showed the presence of homozygous deletion of exon-2 and exon-3 in two patients, two patients showed compound heterozygosity for exon 1 deletion and missense mutation p.E462V as a second allele and one patient was identified with duplication of exon-1 with novel splice site variant c.1527-2A > T as a second allele (Table 2 and Fig. 1). In Silico analysis of the novel variant was identified as disease causing by Mutation taster and NNsplice site 0.9 algorithm.

**Table 2:** MPLA analysis for deletion/duplication study of *HEXA* gene

Patient ID	Deletions/ Duplications	No of Exons Deleted/ Duplicated	^c^ MLPA probe ratio (Dosage quotient)	Clinical relevance
1	Homozygous deletions	2 (Exon 2 and 3)	0.0	Yes
2	Homozygous deletions	2 (Exon 2 and 3)	0.0	Yes
3^a^	Heterozygous deletion	1 (Exon 1)	0.5	Yes
4^a^	Heterozygous deletion	1 (Exon 1)	0.5	Yes
5^b^	Heterozygous duplication	1 (Exon 1)	1.3	Yes

^a^Compound heterozygous with p.E462V as a second allele

^b^Compound heterozygous with c.1527-2A > T as a second allele

^c^>MLPA ratios (dosage quotient) of below 0.7 or above 1.3 are indicative of a deletion (copy number change from two to one) or duplication (copy number change from two to three), respectively. A dosage quotient of 0.0 indicates a homozygous deletion, 0.35 to 0.65 indicates heterozygous deletion, 1.3 to 1.55 indicates heterozygous duplication and 1.7 to 2.2 indicates homozygous duplication

## Discussion

The clinical appearance and neuroimaging features of infantile TSD seen in our patients were consistent with the defined phenotype. All patients presented with the severe infantile form of the disease irrespective of the genotype. The results of enzyme activity measurements (Hex-A expressed as a percentage of Total-Hex activity) varied from 0 to 0.2%. This is consistent with previous observations that infantile TSD patients have values ranging from 0 to 2% [9, 10].

During the course of the analysis we could not identify the second disease-causing allele in three patients and no variant was identified in two patients after sequencing the entire coding region of *HEXA* gene. Among the possible underlying reason for these findings, we suspected the presence of a deletion or duplication in the gene. Therefore using MLPA specific for *HEXA* gene we could identify two large deletions and one large duplication in an Indian TSD patients for the first time that include homozygous deletion of exon 2 & 3 in two cases, compound heterozygous deletion of exon-1 with second founder mutation p.E462V in two cases [6] and compound heterozygous duplication of exon-1 with second novel splice site variant c.1527-2A > T (Table 1). The carrier frequency of p.E462V mutation is ~ 1/500 which was earlier reported by Mistri et al. in 2012 [6]. In addition to this, recently the said variant was also reported only in one South Asian sample (http://gnomad.broadinstitute.org/variant/15-72638612-T-A). As has been known, the 7.6 kb deletion is the major mutation causing TSD in the French Canadian population; it removes part of intron-1, all of exon-1 and extends 2 kb upstream, encompassing the putative promoter region [4]. Although, this deletionwas never identified in our large cohort of Indian patient with TSD. Nonetheless, large deletion encompassing one or two exons or duplication of one exon are never reported and identified as a first disease causing variation in *HEXA* gene so far. Severity of phenotype in all five patients could be explained by the truncation of normal protein structure due to exon deletion/duplication in the gene.

This experimental approach of determination towards quantitative copy number variation in identifying large deletion and/or duplication is novel and reported here for the first time. The present study and earlier publications from our group [6, 7] also demonstrates that Indian TSD patients mainly portray infantile onset with severe phenotype irrespective of the genotype. None of our patients showed juvenile or late onset presentation. Though, it is highly likely that they are missed due to lack of awareness and failure of clinical identification as well. However, there are few mutations that have been identified in the late-onset phenotypes [11].

## Conclusion

The present study demonstrates that large deletion and/or duplication in *HEXA* gene needs to beconsidered as the second tier approach in thegenomic sites where no variants are observed by conventional Sanger sequencing.

## Abbreviations

Hex-A: β-hexosaminidase-A
LSDs: Lysosomal storage disorders
MLPA: Multiplex Ligation-dependent Probe Amplification
TSD: Tay-Sachs disease
